# The Mediport Paradox: Mediastinitis and Pericardial Effusion With a Misplaced Mediport

**DOI:** 10.7759/cureus.24562

**Published:** 2022-04-28

**Authors:** Bilal Malik, Amman Yousaf, Mohammed Berrou, Arvind Kunadi

**Affiliations:** 1 Internal Medicine, McLaren Health Care, Flint/Michigan State University (MSU), Flint, USA; 2 Internal Medicine, McLaren Flint, Flint, USA; 3 Radiology, Services Institute of Medical Sciences, Lahore, PAK; 4 Pulmonary and Critical Care Medicine, McLaren Flint, Flint, USA; 5 Internal Medicine/Nephrology, McLaren Health Care, Flint/Michigan State University (MSU), Michigan, USA

**Keywords:** mediastinitis management, pericardial effusion, cancer immunotherapy, immunotherapy, mediport

## Abstract

Totally implantable subcutaneous devices (TISDs) have become excellent options for patients requiring long-term chemotherapy, parenteral nutrition, and fluid replacement. As with all invasive devices and procedures, they come with their inherent risks, which may manifest immediately or at a later point in time. We present the case of a 74-year-old female with a history of hypertension, chronic obstructive pulmonary disease (COPD), ischemic stroke, breast cancer, and lung cancer who had mediport placement for chemotherapy administration. She received several infusions of pembrolizumab through her mediport and developed progressive dyspnea over four weeks. Upon evaluation at our institution, she was found to have a misplaced mediport with mediastinitis and pericardial effusion due to direct mediastinal exposure to immunotherapy. This case highlights the importance of systematic imaging review, regardless of suspected pathology, and encourages providers to have a low threshold to re-evaluate patients after device placement or immunotherapy commencement.

## Introduction

Totally implantable subcutaneous devices (TISDs) such as mediports are often used for long-term chemotherapy, parenteral nutrition, and fluid replacement. These were first developed in 1980 [1], with almost half a million mediports inserted in the United States annually in the present-day setting [2]. Following standard protocols and appropriate post-insertion imaging evaluation minimizes catheter-related complications, which occur in as many as one-third of cases (across all categories of complications) [3]. Many types of complications such as catheter infection, catheter fracture, pneumothorax, hemothorax, catheter arterial injury, venous perforation, air embolism, and nerve injuries have been reported in association with port placement and utilization [4]. We report the case of an elderly female who developed two rare pembrolizumab-associated complications, mediastinitis, and pericardial effusion, in association with inadvertent extravascular mediport placement and direct mediastinal exposure to immunotherapy.

## Case presentation

A 74-year-old female patient with a past medical history of hypertension, chronic obstructive pulmonary disease (COPD), ischemic stroke, left breast cancer, and left lung non-small cell cancer presented to the emergency department with progressive dyspnea of five weeks duration. Her dyspnea was refractory to her COPD home medications. Her vitals on presentation included a heart rate of 75 beats per minute, respiratory rate of 28 breaths per minute, 93% saturation on 4 L nasal cannula, blood pressure of 99/61 mmHg, and temperature of 98.2 degrees Fahrenheit. Her laboratory studies were significant for a white blood cell count of 13.2 x 103/microliter, hemoglobin of 8.3 g/dL, red cell distribution width of 19.9%, and an absolute neutrophil count of 11.50 x 103/ microliter, but otherwise unremarkable. She was started on oxygen by nasal cannula, IV ceftriaxone, and admitted to the intensive care unit for further workup and management.

**Figure 1:**
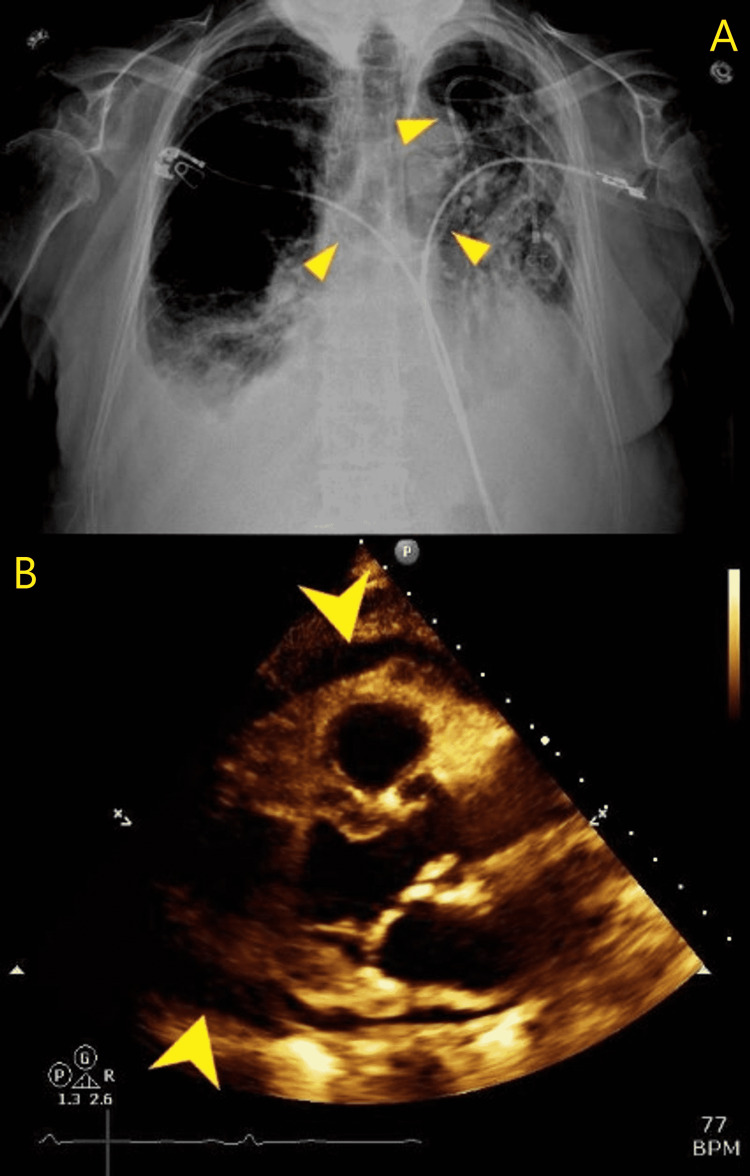
(A) Chest X-ray (AP view) - chest X-ray demonstrating bilateral interstitial infiltrates and the malpositioned tip of the mediport in the right mediastinum (yellow triangles). (B) Long axis echocardiogram view demonstrating pericardial effusion (yellow arrowheads). No tamponade physiology is seen. AP, anteroposterior

**Figure 2:**
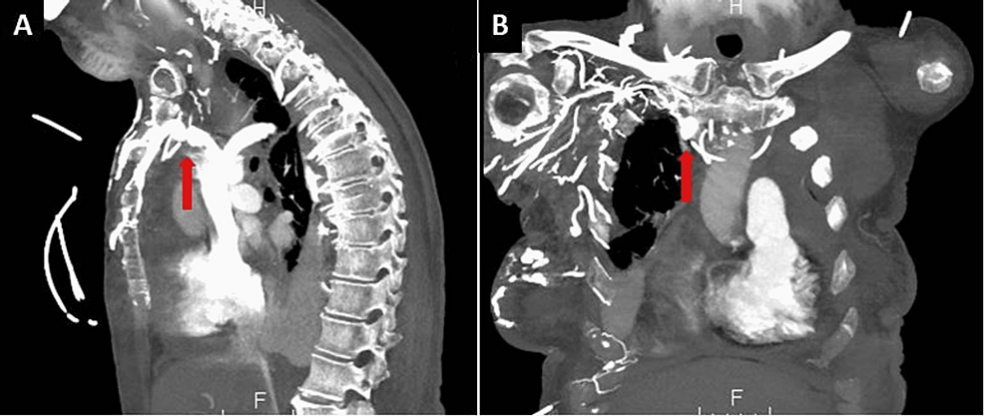
Contrast-enhanced CT scan of the thorax: (A) sagittal view and (B) coronal view demonstrating mediport outside the vascular structures with the tip in the anterior mediastinum, just outside the right brachiocephalic vein (arrows).

In view of her history of breast cancer, she had a previous left-sided partial mastectomy and sentinel lymph nodes dissection. She also subsequently developed lung cancer, for which she received a multidrug chemotherapy regimen that included carboplatin, pemetrexed, and pembrolizumab. After induction, her chemotherapy regimen was de-escalated to pembrolizumab monotherapy through a mediport, which was placed four weeks prior to her presentation. A chest X-ray (CXR) demonstrated bilateral pleural effusion and no parenchymal abnormality (Figure 1A). A CT scan of the chest with contrast was done in view of persistent dyspnoea despite oxygen supplementation. It was negative for pulmonary embolism but did demonstrate mediport placement within the anterior mediastinum in the right hemithorax, adjacent to the right brachiocephalic vein (Figure 2). Contrast extravasation was also evident in the mediastinum with mediastinal fat-stranding and pericardial effusion. Unfortunately, in the preceding weeks leading up to her admission, the patient had received four rounds of pembrolizumab therapy into her misplaced mediport.

With her history of breast and lung malignancies, the possibility of malignant pericardial effusion was raised. Cardiology performed an echocardiogram which revealed pericardial effusion without tamponade physiology (Figure 1B - above). After consultation with cardiothoracic surgery, a decision was made to perform a diagnostic and therapeutic pericardial window. Histopathologic examination of the pericardial fluid confirmed inflammatory effusion. It revealed the absence of malignant cells, acid-fast bacilli, bacterial, or fungal growth, while samples from the pericardial tissue demonstrated myxoid inflammatory changes. The mediport was removed, and unfortunately, at the time of writing the patient remained admitted to our institution for further care.

## Discussion

Modern techniques utilizing ultrasound or CT guidance during the insertions of central venous catheters/devices have a low incidence of post-procedural complications. Several types of complications such as catheter infection, catheter fracture, pneumothorax, hemothorax, catheter arterial injury, venous perforation, air embolism, and nerve injuries have been reported in associations with port placement and utilization [4-5]. The presentation of such complications may vary drastically, from acute hemodynamic compromise in pneumothoraces to no immediate symptomatology in a misplaced port. Malpositioning is routinely ruled out by post-insertion imaging and has become increasingly rare. Appropriate positioning is confirmed by the projection of the tip of the catheter along the superior vena cava (SVC) into the lower third at the junction between the right atrium and SVC [6].

In our patient, the misplaced port was not detected at the time of insertion, post-procedural imaging, or at the time of admission due to dyspnea. Multiple healthcare professionals including pulmonologists, radiologists, and internists, failed to detect the misplaced port. This likely occurred because of the patient’s history and each provider’s focus on a potential problem in the parenchyma of the lungs alone. This oversight reinforces the basics of CXR interpretation, using a systematic approach. The classic “ABCDE” (Airway, Breathing, Circulation, Disability/Devices, Exposure) approach provides a basic system in which to review CXRs [7]. Moreover, physicians should have a low threshold to retrospectively review the imaging or have a CT thorax to avoid delay in diagnosing mispositioned catheters and their associated complications.

Pembrolizumab is IgG-4 humanized antibody that has been used as immunotherapy in various malignancies such as non-small lung cancer, Hodgkin's lymphoma, urothelial, gastric, and head and neck cancers [5]. Malignant cells usually overexpress program death-1 (PD-1), PD-1 ligand, and PD-2 ligands, which help them evade the host immune system. By binding to PD-1 receptors on T-lymphocytes, PD ligands allow malignant cells to proliferate unchecked. Pembrolizumab has a specific affinity for PD-1 receptors on T-lymphocytes, preventing PD-1 receptors from binding to PD-1 and PD-2 ligands and allowing T-cells to appropriately perform their role in the immune response. The activation of this cascade mobilizes T-lymphocytes against malignant cells [6-7].

Various risk factors have been documented to be predictors of immune checkpoint inhibitor toxicity, including a family history of autoimmune diseases, history of infective hepatitis, HIV infection, or concurrent immunotoxic medication. The presence of eosinophilia has also been documented as a predictor of toxicity with the use of such agents [6]. The side effects of pembrolizumab are usually mild, including rash, fatigue, and diarrhea, and management is supportive. Organ-specific toxicities including, but not limited to, nephritis, pneumonitis, colitis, diabetes mellitus, pancreatitis, arthritis, and hepatitis have also been documented [6, 8].

Cardiovascular adverse effects are rare with pembrolizumab and usually include myocarditis, pericarditis, or heart block. To the best of our knowledge, there are only three case reports of pericardial effusion to date, and these were documented in association with systemic administration [6, 8-9] (Table 1). It is crucial to differentiate whether the pleural effusion is due to malignancy or adverse effects of medication in patients receiving such agents. The presence of immune cells in the cytology of the pericardial fluid or progression of malignancy on imaging usually indicates malignant pathogenesis of the effusion. However, if there is exudative effusion with lymphocytic predominance in the absence of malignant cells, it is more suggestive of an immune-mediated effusion secondary to the use of immune checkpoint inhibitor toxicity [6, 8]. Upon assessment of our patient’s presentation and subsequent pericardial fluid analysis, it is likely that her effusion was secondary to mediastinal pembrolizumab exposure as opposed to a systemic adverse effect.

**Table 1:** A review of previously documented cases of pericardial effusion associated with pembrolizumab use.

Case report	Oristrell et al. [10]	Li et al. [11]	Atallah-Yunes et al. [8]	Malik et al. [this report]
Year of publication	2018	2018	2019	2021
Primary malignancy	Infiltrating ductal carcinoma of the left breast	Non-small cell lung carcinoma	Metastatic left lung squamous cell carcinoma	Left breast cancer, Left lung non-small cell carcinoma
Comorbidities	Tobacco smoking	HIV infection-adequately treated with viral load undetectable, Stable CD-4 count	Tobacco smoking, atrial fibrillation, vocal cord paralysis, emphysema, and hypertension	Essential hypertension, chronic obstructive pulmonary disease, ischemic stroke
Presenting signs and symptoms	Tachycardia, chest pain, hypotension	Palpitations	Progressive dyspnea	Progressive dyspnea
Other drugs in the treatment regimen	Nab-paclitaxel, carboplatin, adriamycin	Carboplatin/pemetrexed, palliative radiotherapy for bone metastasis	None was a candidate for chemotherapy	Carboplatin, pemetrexed
Duration of pericardial effusion after the start of pembrolizumab	Six months after completing the treatment	Three months/ after the third cycle	One week after the first dose of pembrolizumab	Three weeks after the patient was solely on pembrolizumab
Accompanying side effect	Adrenal Insufficiency	None	None	Mediastinal inflammatory changes
Management and recurrence	Diagnostic pericardiocentesis was done, pericardial effusion recurred, and then started on a steroid to which the patient responded well.	Started on Prednisolone.	Pembrolizumab discontinued, pericardial drain placed, started on Prednisolone	Pericardiocentesis/ pericardial window started on prednisolone
Outcome	Complete resolution with no recurrence on six months follow-up.	The patient died after three months	The drain was removed after three days after the output was significantly reduced. The patient was discharged on a tapering dose of steroids	Complete resolution of mediastinitis and pericardial effusion with no recurrence

According to the American Society of Clinical Oncology, the management of immune checkpoint inhibitor-induced pericardial effusion is the discontinuation of the causative agent and starting the patient on high-dose corticosteroids at 1-2 mg/kg dosing [10]. Importantly, our patient did receive direct mediastinal exposure to a medication that was not intended for soft tissue administration. This exposure, regardless of the agent, would likely cause mediastinitis and inflammatory reaction as a result of soft tissue administration. In our patient, considering the faulty placement of the mediport and her subsequent mediastinitis and pericardial effusion, steroids were not utilized due to the effusions and mediastinitis being the result of a direct insult to the respective spaces.

## Conclusions

We presented the case of a 74-year-old female patient with a past medical history of COPD, ischemic stroke, left breast cancer, and left lung non-small cell cancer who presented to the emergency department with progressive dyspnea. She was identified to have a misplaced mediport and resulting mediastinitis, and pericardial effusion due to direct mediastinal exposure to the immunotherapy. This case highlights the importance of having a systematic approach to imaging and having a low threshold for re-assessment of patients following implantation of therapeutic devices or commencing novel immunologic therapies.
